# Comparison of Real-Life Systems of Care for ST-Segment Elevation Myocardial Infarction

**DOI:** 10.5334/gh.343

**Published:** 2020-10-01

**Authors:** Surya Dharma

**Affiliations:** 1 Department of Cardiology and Vascular Medicine, Faculty of Medicine, University of Indonesia, Indonesian Cardiovascular Research Center, National Cardiovascular Center Harapan Kita, Jakarta, ID

**Keywords:** STEMI, system of care, reperfusion therapy, health care infrastructure, guidelines recommended therapy

## Abstract

The success of ST-segment elevation myocardial infarction (STEMI) networks application in Europe and the United States in delivering rapid reperfusion therapy in the community have become an inspiration to other developing countries to develop regional STEMI network in order to improve the STEMI care. Although barriers are found in the beginning phase of constructing the network, recent analysis from national or regional registries worldwide have shown improvement of the STEMI care in many countries over the years. To improve the overall care of patients with STEMI particularly in developing countries, improvements should be focusing on how to minimize the total ischemia time, and this includes care improvement at each step of care after the patient shows signs and symptoms of chest pain. Innovation in health technology to develop the electrocardiogram transmission and communication system, along with routine performance measures of the STEMI network may help bridging the disparities of STEMI system of care between guideline recommended therapy and the real world clinical practice.

## Introduction

The success of ST-segment elevation myocardial infarction (STEMI) network introduction in Europe and the United States (US) in increasing the utilization of rapid reperfusion therapy in the community has become an inspiration for many countries to construct a national or regional STEMI network in order to improve the outcome of STEMI patients [1,2,3]. Although some barriers are found in the beginning phase of constructing a STEMI network, recent analyses from the national and regional registries that were performed in the high-income and lower-to-middle income countries have shown improvement of care for STEMI patients over the years [4,5,6,7].

Variables that affect early reperfusion therapy in the community including the availability of the health care infrastructures that may varies between each region or country (Table 1) [8], and can be identified by analyzing the data from the on-going national or regional registries as part of the STEMI network program, the so-called performance measures [9]. The results of the analysis are often used as feedback to improve the performance of the STEMI network.

**Table 1:** Barriers for a rapid reperfusion therapy in application of the STEMI network.


Geographic Logistics Administration process Ambulance equipment Traffic control ECG transmission system Catheterization laboratory facilities Human resources Local culture Health insurance reimbursement policy Health technology


## Methodology

A comprehensive overview of the STEMI network organization is needed to study the achievements and challenges at each step of care of patients with STEMI after the patients showed sign and symptom of ‘heart attack’. In this review, the different type of real-life STEMI systems of care in developed and lower-to-middle income countries are described, and gives insights to current concept of management and areas for improvement. Current literatures on STEMI networks in developed and lower-to-middle income countries were reviewed and summarized in this review. Additional relevant articles searches were performed in MEDLINE and PUBMED using relevant search words included STEMI, system of care, reperfusion therapy, developing/developed countries, barriers/challenges for reperfusion therapy, total ischemia time, primary percutaneous coronary intervention (PCI), door-in to door-out (DI-DO) time, and door-to-device (DTD) time. Articles searches were also made using the continent names (Asia, Africa, America, Europe, and Australia).

This review article reflects the view of the author in some of the suggestions to improve the STEMI care in several regions, and that others may have difference views. Therefore, the goal of this review is to stimulate discussion among the STEMI network stakeholders.

## Historical Perspective

The identification of thrombus in occluded coronary arteries and the introduction of mechanical coronary recanalization in the early ’80s have made a substantial change in the way of treating patients with acute STEMI [10,11]. The first administration of thrombolytic therapy in acute myocardial infarction (AMI) by Chavoz et al. in 1976 was applied into wider clinical practice after the publication of randomized clinical trials GISSI and ISIS-2 [12,13,14]. The first use of primary angioplasty in AMI by Meyer et al. in 1982 and by Hartzler et al. in 1983 were followed by the first three randomized clinical trials in 1993 showing superiority of primary PCI over thrombolytic therapy in STEMI [15,16,17,18,19]. Ten years later (2003), the superiority of primary PCI over thrombolytic therapy in reducing the mortality rate of STEMI patients was further confirmed by the results of a meta-analysis of 23 randomized clinical trials [20]. However, in the real world setting, not all patients with STEMI can be treated by a timely primary PCI due to several barriers [21,22,23]. To overcome the barriers for a rapid reperfusion therapy in the community, a regional STEMI network is introduced to organize care between non-PCI hospitals, ambulance and PCI centers in order to deliver a rapid reperfusion therapy in the community as recommended by the AHA and ESC guidelines [24,25].

## Pre-Hospital/Community Setting

The ischemic time counts from the start of chest pain. Therefore, patients should aware on the sign and symptom of heart attack, the medical emergency phone number, and know where to go for seek help and/or call an ambulance. To increase the awareness of the community, a public campaign on the management of chest pain should be performed, and has been shown to be associated with a significant reduction in treatment delay for patients with AMI (ranging from 42 min to 52 minutes of reduction time) [26,27].

## Non-PCI Center Setting

The processes of care at the non-PCI center starts from the arrival of the patient at the emergency department (ED) of the hospital to the initiation of reperfusion therapy. This included the time of admission-to-first medical contact (FMC), FMC-to-ECG recording, ECG recording-to-STEMI diagnosis, and STEMI diagnosis-to-reperfusion therapy [25]. The choice of reperfusion therapy in a non-PCI center is either fibrinolytic therapy (targeted door-to-needle time of <30 minutes), or immediate transfer to a PCI center for primary PCI (targeted STEMI diagnosis-to-wire crossing time of <120 minutes) [25]. The performance of treating STEMI patients at non-PCI center can be measured by the DI-DO time, which is the time spent by the patient at the initial hospital before being transferred to a primary PCI center for primary PCI. Longer DI-DO time is associated with incremental increased odds of mortality [28].

## PCI center setting

The performance of the PCI center in treating STEMI patients is measured by the achievement of the DTD time <60 minutes [25]. This include several care steps from ED admission at the PCI center, catheterization laboratory activation, transfer time to the catheterization laboratory, and the PCI procedural characteristics (arterial access choice and device selection).

The management of patients with STEMI requires a multi-holistic approach that needs to be standardized by a system of care to minimize the time delays for reperfusion therapy at each step of care. The barriers and challenges of STEMI systems of care application are different between lower-to-middle and high-income countries and deserve further detailed discussion. The differences in methods of reperfusion therapy from various STEMI networks are summarize in Table 2.

**Table 2:** Characteristic of reperfusion therapy from various STEMI networks.

Country	Current achievement of reperfusion therapy	Source of data

Europe		
France, (N = 14,423)	Use of primary PCI increased from 12% in 1995 to 76% in 2015.	FAST-MI registry
Vienna, (N = 1053)	Two years after STEMI network introduction, reperfusion therapy increased from 66% to 87%, and the proportion of non-reperfused patients reduced from 34% to 13.4%.	Vienna STEMI registry
United States, (N = 147,466)	– Improvement of DTD and DI-DO times in 2012 compared with 2008 (median 59 min versus 68 min, and median 62 min versus 76 min, respectively) – Use of fibrinolytic therapy and non-reperfused patients declined in 2012 compared with 2008 (7% versus 13.4% and 3.3% versus 6.2%, respectively).	Mission: Lifeline programme
Russia, (N = 85,496)	Use of primary PCI and fibrinolysis therapy were 24% and 27.6 %, respectively.	Russian Acute Coronary Syndrome Registry
Australia, (N = 4110)	Rate of primary PCI was not increase over time but access to non-PCI center was increasing.	
Asia		
India (southern state of Tamil Nadu), (N = 2420)	A hub-and-spoke model improved STEMI care by higher utilization of primary PCI.	Local registry
China, (N = 13,815)	Use of primary PCI increased from 10.6% in 2001 to 28.1% in 2011.	China PEACE-Retrospective Acute Myocardial Infarction Study
Indonesia (Jakarta), (N = 1676)	– Use of primary PCI increased from 28% in 2008/2009 to 56% in 2015/2016. – the median DTD time improved from 94 min to 82 min.	Jakarta Acute Coronary Syndrome Registry
Korea, (N = 32,211)	– The symptom onset-to-balloon time has gradually decreased from 257 min in 2008 to 189 min in 2018. – The door-to-balloon time reduced from 72 min in 2008 to 60 min in 2012, and remained at approximately 60 min since 2012 to 2018.	Korea Acute Myocardial Infarction Registry
Japan, (N = 20,462)	– Rates of ambulance use and primary PCI were 78.9% and 87.9%, respectively. – The median time from symptom onset-to-balloon time and median door-to-balloon time were 230 min and 80 min, respectively.	Japan Acute Myocardial Infarction Registry
Singapore, (N = 4667)	– Less than half of STEMI patients (49.8%) utilized EMS transport. – Patients who used EMS transport were associated with higher rate of reperfusion therapy, and resulted to shorter median symptom onset-to-balloon and door-to-balloon times.	Singapore Myocardial Infarction Registry
Middle East, (N = 2233)	Use of fibrinolysis therapy and primary PCI were 29% and 42.5%, respectively.	Saudi Acute Myocardial Infarction Registry
Latin America		
Brazil, (N = 520)	Use of telemedicine in a regional STEMI network increased primary reperfusion procedures (53.8% versus 29.1%), and more patients transferred to referral hospitals (76.3% versus 44.7%).	Salvador’s STEMI registry (RESISST)

PCI denotes percutaneous coronary intervention; STEMI, ST-segment elevation myocardial infarction; DTD, door-to-device; DI-DO, door-in to door-out; EMS, emergency medical service; N indicates number of patients included in the study.

## STEMI networks in developed countries

### Europe

Most of the STEMI networks protocols in European countries are concordance with the ESC guideline for STEMI [25]. In STEMI networks of North, West and Central European countries, primary PCI is used as the preferred reperfusion strategy for the majority of STEMI patients. Variables that are affecting the reperfusion choices are the distribution of 24/7 PCI facilities and transfer time that found to be different among the STEMI networks of the European countries [29]. The examples of successes STEMI networks organization in Europe can be studied from the STEMI network organized in France and Vienna, Austria.

### France

The Service d’Aide Medicale Urgente (SAMU) network is an example of a well-established STEMI system of care in Europe that successfully organized the care of patients with STEMI across the nation by making coordination between non-PCI center, ambulance and PCI center to provide rapid transfer for primary PCI. The SAMU network was started in 1995 with a unique nationwide call number and has a physician-staffed ambulance on site. A pharmaco-invasive strategy is adopted in the protocol, whereas 96% of patients who received fibrinolysis had coronary angiogram [30]. The French registry of Acute ST-segment elevation or non-ST-segment elevation Myocardial Infarction (FAST-MI) evaluated all patients hospitalized for acute MI in France, and has been used routinely as the performance measures of the STEMI network in France since 1995 [31,32]. During 20 years observation (1995 to 2015), use of primary PCI in France increased from 12% (1995) to 76% (2015), and the 6-month mortality constantly decreased from 17.2% in 1995 to 6.9% in 2010 and 5.3% in 2015 [5].

### Vienna

The STEMI network in the city of Vienna, Austria was started in March 2003 [33]. The initiative to optimize the reperfusion strategy for STEMI (primary PCI or thrombolytic therapy) was organized by the Viennese Ambulance system and a network of catheterization laboratories located in Vienna. The Vienna STEMI registry applied extensively in the region is being used as the performance measures of the STEMI network. The registry data showed that two years after introduction of the STEMI network in Vienna has resulted in an increased proportion of patients who receive reperfusion therapy (from 66% to 87%), and a reduced proportion of non-reperfused patients (from 34% to 13.4%) [30].

## Trends in primary PCI in European STEMI networks (2010 to 2015)

In general, use of primary PCI in several European countries increased consistently in the year 2010 to 2015 (Table 3) [34]. However, the uptake of primary PCI was higher in countries like Egypt, Kazakhstan, Macedonia, Portugal, and Serbia (Group A) compared with the uptake of primary PCI in Belgium, Denmark, France, Israel, Italy, Poland, Spain, Sweden, Switzerland and United Kingdom (Group B). This leads to a higher percentage of primary PCI to the total numbers of PCI in countries of Group A than Group B (~20–30% vs. ~8–20%) [34]. The exact reason for the higher uptake of primary PCI in Group A is probably related to the increasing gross national income in Group A countries over the years with improved health care infrastructure.

**Table 3:** Trends in primary PCI in European STEMI networks. [34].

Countries	2010	2011	2012	2013	2014	2015

Belgium	NA	4,365	NA	4,088	4,399	4,817
Denmark*	309	390	403	400	404	433
Israel	1,574	1,640	1,780	1,773	1,802	1,820
Italy	27,908	28,514	30,038	31,957	32,557	33,895
Kazakhstan	347	365	1,180	1,694	1,886	2,368
Macedonia	763	747	735	1,001	1,291	1,175
Poland	25,634	28,060	28,278	26,681	26,678	30,163
Portugal	1,773	2,230	2,952	3,155	3,121	3,267
Serbia	2,676	3,493	3,834	4,239	4,743	5,093
Spain	10,339	11,766	13,690	13,890	14,679	15,089
Sweden	4,646	4,559	4,576	4,666	4,929	4,902
Switzerland	3,985	3,639	3,139	3,084	3,393	3,825

Data are presented as number of PCI procedures; NA, not available. * Data are presented as number of procedures per million inhabitants.

## STEMI network in the United States (US)

In the US, 79% individuals are within <60 minutes of driving time to a PCI center and 34% of non-PCI centers are located within 30 minutes of driving time to the PCI centers [35], suggesting that the majority of STEMI patients in the US should be treated with primary PCI within the guideline recommended time. Data from the Mission: Lifeline programme (147,466 patients from 485 hospitals) showed improvement of DTD and DI-DO times in 2012 compared with 2008 (median 59 minutes versus 68 minutes, P < 0.001; and median 62 minutes versus 76 minutes, P < 0.001, respectively). Use of fibrinolytic therapy and non-reperfused patients declined in 2012 compared with 2008 (7% versus 13.4% and 3.3% versus 6.2%, respectively). Furthermore, the proportion of patients who presented directly to PCI center via emergency medical system (EMS) increased in 2012 compared with 2008 (43% versus 38.4%) [36]. These data suggest that the Mission: Lifeline programme introduced in the community resulted in improvement of STEMI care in the US from 2008 to 2012. Further action is still needed to minimize the time delay for primary PCI by means of a DI-DO time of <30 minutes should be achieved in the majority of patients who first presented to a non-PCI hospital, as recommended by the AHA guideline [24].

## STEMI networks in India, China, Indonesia and Russia

Barriers for a rapid reperfusion therapy in countries with rapidly growing epidemic of cardiovascular disease including overcrowded urban centers, long transfer delays, patient arrive to ED by public/private transport, and lack of EMS organization and insurance system [23]. The data from 2,420 patients with STEMI in the southern state of Tamil Nadu in India showed that a hub-and-spoke model improved STEMI care by higher utilization of primary PCI and was associated with a reduced 1-year mortality [37]. The findings suggest that the hub-and-spoke model is visible and can be extensively introduced in other region in India.

In China, the analysis from 13,815 patients with STEMI during 2001–2011 showed that hospital admissions for STEMI have risen over time. Although the use of primary PCI increased from 10.6% in 2001 to 28.1% in 2011 (P_trend_ < 0.0001), the percentage of patients who did not receive reperfusion therapy did not change (45.3% in 2001 vs. 44.8% in 2011, P_trend_ = 0.69). The adjusted in-hospital mortality was also not changed (odds ratio = 0.82, 95% confidence interval 0.62 to 1.10, P_trend_ = 0.07) [38].

The China STEMI care Project 2019 was recently launched in 18 provinces as an integrated regional STEMI network with the aim to increase the use of reperfusion therapy in China. This project is planned for 10 years. The pharmaco-invasive strategy is adopted in the protocol when STEMI patient is admitted to a non-PCI hospital, followed by direct transfer to a PCI center [39]. It is expected that the results of the project will likely to improve the care of STEMI patients in the region.

Other example of a STEMI network in developing country can be studied from the Jakarta Cardiovascular Care Unit Network System that was introduced in Jakarta, Indonesia in 2010 [40]. The regional STEMI network was developed and hosted by the ED of a tertiary care academic hospital located in the metropolitan area of Jakarta using WhatsApp, faximile and email as methods for ECG transmission. Half a decade after implementation of the regional STEMI network, more primary PCI procedures were performed in the hospital (56% vs. 28%, P < 0.001), shorter DTD time for primary PCI was achieved (median 82 vs. 94 minutes, P < 0.001, fewer patients did not receive reperfusion therapy [37% vs. 59%, P < 0.001]), and the in-hospital mortality reduced by 26% [7]. The STEMI network in Jakarta is currently being expanded to the surrounding area of Jakarta to cover ~26 million inhabitants.

In Russia, the trends in PCI procedures have increased in all districts during the period 2005 to 2013 [41]. Data from the Russian Acute Coronary Syndrome Registry from 2008 to 2015 consist of 85,496 patients with STEMI and showed that the use of primary PCI and fibrinolysis therapy were 24% and 27.6 %, respectively [42]. The data suggest that almost half of STEMI patients did not receive acute reperfusion therapy. The wide application of the STEMI network protocol along with wide adoption of the registry is needed to improve the care of STEMI patients in the whole country.

### Australia

In 2006, the population in Australia consists of 14.5 million adults with 351 acute care hospitals, of which 42 hospitals (12%) have primary PCI service. A pharmaco-invasive strategy is widely adopted in the region [43]. Recent report from Australia in an observation of 4,110 patients with STEMI who admitted between 1999 and 2016 showed that the rate of primary PCI was not increase over time but access to non-PCI center was increasing [44]. The findings encouraged a wider uptake of fibrinolytic therapy at the non-PCI center, followed by direct transfer of the patient to a nearest PCI center for coronary angiogram evaluation.

## STEMI Systems of Care in Other Low-to-Middle-Income Countries

The majority of other developing countries used fibrinolysis therapy as the method of reperfusion therapy in the community such as in Algeria (49%), Kenya (79.5%), Libya (54%), Tunisia (49%), Iran (46.3%), and Bangladesh (81.2%). The reported door-to-needle time for fibrinolytic therapy in those countries did not reach the guideline recommendation (<30 minute) in the majority of cases [45]. The data suggest that the utilization of primary PCI in many developing countries remains low, and efforts are needed to improve the STEMI care in those countries in order to achieve a faster and better care by probably improving the health care infrastructures.

### Middle East

The first survey of the Saudi Acute Myocardial Infarction Registry program during 2015–2017 included 2,233 patients with ACS, of which 65.9% were STEMI. The survey showed that emergency medical service (EMS) was utilized in only 5.2% of cases. Use of fibrinolysis therapy and primary PCI were 29% and 42.5%, respectively [46]. The data suggest efforts are needed to organize a STEMI network program in the whole region using common protocols that are recommended by the guidelines. Such efforts include the wide use of EMS and direct transfer for primary PCI in the majority of STEMI patients.

### Latin America (Brazil)

Data from Brazil identified several barriers for rapid reperfusion in STEMI care including delay from symptom onset-to-ED admission, use of self transportation to hospital admission in such proportion of patients, and delay from ED admission to first ECG recording [47]. To improve the care of STEMI patients in the whole region in Brazil, implementation of a regional STEMI network supported by telemedicine and utilizing local pre-hospital EMS might be the best solution. This system of care was associated with lower mortality and higher use of evidence-based therapies, as suggested by a recent analysis from Brazil [48].

### South Africa

Up to 72% of South Africans live within two hours of driving time from a PCI center. However, there are several barriers identified for a timely primary PCI in South Africa including many patients are still using self transportation to hospital admission, and lack of health insurance system and EMS organization [49]. There is a strong need to build a STEMI network in the region that may organize the care of the STEMI patients at the non-PCI and PCI center including improvement of EMS, and ambulance coordination. Other strategy probably increasing the use of fibrinolytic therapy (as part of a pharmaco-invasive strategy) if the patient admitted to a non-PCI center, improvement of health insurance system and educate the people to call the ambulance/EMS phone number if a sign and symptom of heart attack is suspected.

### Korea

The Korea Acute Myocardial Infarction Registry studied 32,211 patients with STEMI in Korea and found that the symptom onset-to-balloon time for STEMI patients treated by primary PCI has gradually decreased from 257 minutes in 2008 to 189 minutes in 2018. The door-to-balloon time reduced from 72 minutes in 2008 to 60 minutes in 2012, and remained at approximately 60 minutes since 2012 to 2018. The authors suggest that educational programs on the manifestation of STEMI are needed to minimize out-of-hospital delays [50].

### Japan

The Japan Acute Myocardial Infarction Registry is a nationwide registry in Japan that included 20,462 patients with acute myocardial infarction between 2011 and 2013, of which 79.7% were STEMI. The data showed that rates of ambulance use and primary PCI were 78.9% and 87.9%, respectively. In the overall population, the median time from symptom onset-to-balloon time and median door-to-balloon time were 230 minutes and 80 minutes, respectively. The percentage of patients who admitted to the hospitals by self-transportation was 18.3%. The overall in-hospital mortality was 8.3% [51].

Despite the high use of ambulance and primary PCI for STEMI patients in Japan, efforts are still needed to minimize the symptom onset-to-balloon time in order to improve the outcome of STEMI patients by probably continuous education for people to use ambulance/EMS for hospital admission, along with improvement of door-to-balloon time to <60 minutes in the PCI center, as recommended by the guideline [25].

### Singapore

Data from the Singapore Myocardial Infarction Registry included 4,667 patients with STEMI enrolled from 2010 to 2012 and showed that less than half of STEMI patients (49.8%) utilized EMS transport. Patients who used EMS transport were associated with higher rate of reperfusion therapy and resulted to shorter median symptom onset-to-balloon and door-to-balloon times [52]. The findings suggest that the STEMI network in Singapore should include programs that educate the community to call the ambulance/EMS using a unique phone number as a mode of transportation to a PCI center when a symptom of heart attack is suspected.

## DI-DO Time in Developed and Developing Countries

**Figure 1:**
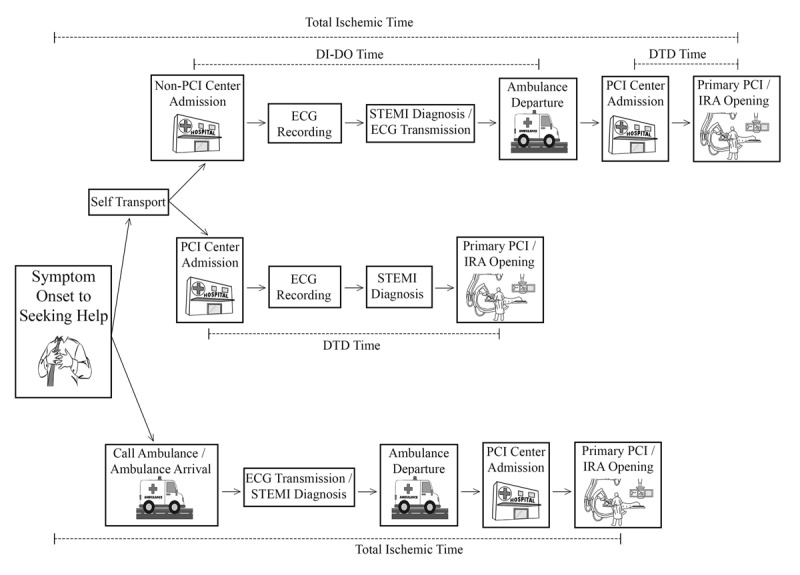
**STEMI chain of survival.** PCI, percutaneous coronary intervention; DI-DO, door-in to door-out; DTD, door-to-device; ECG, electrocardiography; STEMI, ST-segment elevation myocardial infarction; IRA, infarct-related artery.

The performance of treating patients with STEMI at the non-PCI center can be measured by evaluating the time spend by the patient at the non-PCI center before being transferred to a PCI center, the so-called DI-DO time (Figure 1). The DI-DO time in developed country can be studied from the US data. The AHA guideline has recommended a DI-DO time of <30 minutes [24]. However, the real world data from the US showed that among 14,821 patients with STEMI transferred for primary PCI, the majority of patients had DI-DO time between 31 to 90 minutes [28]. Although the reported median DI-DO time in 2012 has improved to 62 minutes [36], but it was still beyond the guideline recommendation of <30 minutes.

In developing country, the data on DI-DO time is very limited. Data from Jakarta Acute Coronary Syndrome registry in Jakarta, Indonesia showed that among 1,076 patients with STEMI transferred for primary PCI in the metropolitan area of Jakarta, the median DI-DO time was 180 minutes (25th percentile to 75th percentile: 120–252 minutes), and the DI-DO time had a positive correlation with the prolonged total ischemia time (r = 0.4 p < 0.001) [53]. DI-DO time is an important ischemic time metric for STEMI patients and untimely DI-DO intervals have been associated with higher in-hospital mortality [28], thus DI-DO time should be improved whenever possible.

## Improving the Regional STEMI Network

### System-related delay

Reports from well-developed STEMI network showed an improvement of DTD time at the PCI center over the years [36]. However, a number of STEMI patients still first admitted to a non-PCI center [36,50], To achieve a timely reperfusion therapy for patients with STEMI in the whole population, care improvement should also be made at the non-PCI centers where the patients first seek help. In this case, the DI-DO time is likely to be an important ischemic time metric in determining the overall delay to primary PCI that correlates with total ischemia time [53]. Interventions aimed at improving DI-DO time in the STEMI networks of both developed and developing countries are likely to have impact on reducing the DI-DO time, that in turn, reducing the total ischemia time (Figure 1). The interventions can include training the emergency physician and nurse who work at the non-PCI center, along with endorse rapid ECG transmission and transfer decision, rapid ambulance coordination, and perhaps introduce fibrinolytic therapy when delay for transferring the patients for primary PCI is highly suspected. In PCI center, future attempts to improve the primary PCI delays included by-passing the ED process to transport the patients directly to the catheterization laboratory is an interesting concept, and if applied then the pre-PCI center care plays a central role to further minimize the total ischemic time.

### Patient-related delay

Patient-related delay that contributes to a longer total ischemia time in a STEMI network is the delay of time frame from symptom onset-to-ED admission (Figure 1). The real-world data from US in 2012 showed that only 43% patients with STEMI presented to PCI centers via EMS [36]. Furthermore, the analysis from 70,093 patients with STEMI admitted to PCI centers within 12 h of symptom onset and treated with PCI showed that patients who admitted directly to a PCI center were associated with a shorter median total ischemia time and lower propensity-matched 12-month mortality compared with patients who admitted to the PCI center through inter-hospital transfer (228 min versus 270 min, P < 0.001, and 9.6% versus 10.4%, P < 0.001, respectively) [54]. Together, the findings suggest that it is important to educate the public to directly find a PCI center if a symptom of heart attack is suspected in order to improve the clinical outcome of the patient. The public campaign should be part of the STEMI network program, and should extensively performed in both developed and developing STEMI networks.

### Human resources

Each country has large different healthcare expenditures including differences in the number of cardiovascular health professionals, educational programmes in cardiology, levels of health spending, and available infrastructure, leading to different access to primary PCI procedures.

The number of cardiologists per million people in the ESC member countries averaged 86.3 (median 72.8) [55]. The number (median 72.8) is relatively high compared to, for example, the number of board-certified cardiologists per million people in a developing country (Indonesia) that was 2.74, as of the 2010 national surveillance [23]. Furthermore, as of data in 2014, the average number of cardiologists per million people in Belgium was relatively high compared with Jakarta (104.5 versus 18.9), although the number of populations was quite similar (~11 million inhabitants) [23,55].

Increasing the number of cardiologist and/or interventional cardiologists is probably the priority in countries with relatively low cardiologist per million people in order to improve the overall patient care.

## Health-Care Related Issues

Several health-care related issues that are also affecting the performance of the regional STEMI network included the local culture, health insurance reimbursement policy, and health technology that may potentially differs among rural and urban areas of each region. The STEMI care can be challenged in areas with limited access to medical services. Several studies have been undertaken to study the possible urban-rural disparities in healthcare resources for the treatment of STEMI patients. A cross sectional study of all STEMI patients admitted to hospitals in the US between 2007 and 2014 (1,551,113 patients) showed lower mortality rate among STEMI patients who admitted from rural areas in the Northeast and urban areas in the Midwest, and the mortality was not associated with household income [56]. In other study, the national data from China found substantial gaps in quality of care in both urban and rural areas [57], suggesting that healthcare improvement should be undertaken in both urban and rural areas.

The barriers from the local culture and health insurance reimbursement policy that potentially affect the performance of treating patients with STEMI can be overcome by an intensive collaboration between all health care stake holders and the local government. Finally, innovation in health technology can bridge the gap in the number of specialists in many countries by probably developing technology that increases the use of timely ECG transmission and communication system between several non-PCI centers, ambulance and a PCI center in each region.

## Conclusion

A tremendous progress has been made in the treatment of patient with STEMI during the last five decades. Data from national and regional registries showed that both high-income and low-to-middle income countries have improved their STEMI systems of care. However, there is still a gap in the STEMI care between those of developing and developed countries. To improve the overall care of patients with STEMI particularly in developing countries, improvements should be focusing on how to minimize the total ischemia time, and this include care improvement at each step of care after the patient shows sign and symptom of chest pain. If the patient first admitted to the non-PCI center, the DI-DO time at the non-PCI center should be minimizes whenever possible, followed by direct transfer of the patient to the nearest PCI center for primary PCI. Public campaign program should be part of the STEMI network program, and is needed to educate people to directly find a PCI center if a symptom of heart attack is suspected, as this approach is associated with shorter total ischemia time and lower one-year mortality compared to inter-hospital transfer. Other variables that affect the timely reperfusion therapy in the community such as insurance reimbursement system, cultural and geographical variations, EMS structures, ECG transmission system, the number of cardiologist/interventional cardiologist and PCI hospitals should be improved by a professional collaboration between the national cardiac society, local government and health care authorities. Finally, innovation in health technology for developing the ECG transmission and communication system, along with routine performance measures of the STEMI network may help in bridging the disparities of STEMI system of care between guideline recommended therapy and the real world clinical practice.
